# Challenging the significance of SUV-based parameters in a large-scale retrospective study on lung lesions

**DOI:** 10.1186/s40644-024-00807-3

**Published:** 2024-11-26

**Authors:** Cristiano Pini, Margarita Kirienko, Fabrizia Gelardi, Paola Bossi, Daoud Rahal, Luca Toschi, Gaia Ninatti, Marcello Rodari, Giuseppe Marulli, Lidija Antunovic, Arturo Chiti, Emanuele Voulaz, Martina Sollini

**Affiliations:** 1 Nuclear Medicine, IRCCS San Raffaele Hospital, Milan, Italy; 2 School of Medicine and Surgery, University of Milano-Bicocca, Monza, Italy; 3 Fondazione IRCCS Istituto Nazionale Dei Tumori, Milan, Italy; 4 Department of Biomedical Sciences, Humanitas University, Via Rita Levi Montalcini, 4, 20072 - Pieve Emanuele, Italy; 5 Faculty of Medicine, Vita-Salute San Raffaele University, Milan, Italy; 6 Department of Pathology, IRCCS Humanitas Research Hospital, Milan, Italy; 7 Medical Oncology and Haematology Unit, IRCCS Humanitas Research Hospital, Milan, Italy; 8 Nuclear Medicine Unit, IRCCS Humanitas Research Hospital, Milan, Italy; 9 Thoracic Surgery Unit, IRCCS Humanitas Research Hospital, Milan, Italy

**Keywords:** Standardised uptake value, Positron emission tomography, [^18^F]FDG, Lung lesions, Prognosis, Biomarker

## Abstract

**Background:**

Although many well-known factors affect the maximum standardized uptake value (SUVmax), it remains the most requested and used parameter, especially among clinicians, despite other parameters, such as the standardized uptake value corrected for lean body mass and the metabolic tumor volume, being proven to be less sensitive to the same factors, more robust, and eventually more informative. This study intends to provide robust evidence regarding the diagnostic and prognostic value of SUVmax in a large cohort of subjects with suspected malignant lung nodules imaged by [^18^F]FDG PET/CT.

**Materials and methods:**

We performed a retrospective analysis of patients with suspected/confirmed primary lung tumours undergoing [18F]FDG PET/CT. The sample size was 567 patients. Demographics, imaging, surgical, histological, and follow-up data were collected. SUVmax was analysed according to histology, stage, scanner, and outcome. The impact on measured values of different reconstruction protocols was assessed. All potential predictors of patients’ outcome were assessed.

**Results:**

91% cases were primary lung tumours. Lung benign nodules or metastases accounted for 5% and 4% of cases. Most patients presented with adenocarcinoma (70%) and stage I disease (51%); 144 patients relapsed and 55 died. SUVmax failed to effectively differentiate benign lesions from primary tumours or metastases. Stage I patients presented lower SUVmax. SUVmax significantly correlated with patient weight, injected [^18^F]FDG activity, and lesion size and differed between reconstructions’ protocols. Survival analyses revealed no independent prognostic significance for SUVmax in progression-free after adjusting for other variables. SUVmax correlated with overall survival, disease stage and tumour histotype.

**Conclusion:**

Our study confirms that SUVmax, though widely employed, present relevant limitations in discriminating between benign lesion and lung cancer, in classifying cancer histotypes, and in predicting patient outcomes independently. Known influencing factors significantly impact on numerical values, thus SUV values should be regarded with caution in clinical practice.

**Supplementary Information:**

The online version contains supplementary material available at 10.1186/s40644-024-00807-3.

## Introduction

The first applications of positron emission-based images to measure cerebral flow date back to the 1940s. The introduction of the “molecular coincidence detection” into a modified single photon emission computed tomography scanner alongside growing availability of biology-tracking radiopharmaceuticals has designated nuclear medicine as a leading discipline to study, diagnose and prognosticate diseases, as announced by the cover of the Journal of Nuclear Medicine in the April 1991 [1].One of the distinctive features of positron emission tomography (PET) imaging is the capability to provide “functional” real-time in-vivo information and measurements. Quantitative image-derived indices have always represented the worth of PET imaging: facilitating immediate understanding, objective communication, and unbiased comparison [2].

The maximum standardised uptake value (SUVmax) which measures the metabolic activity within a lesion, is the most used parameter in PET imaging since 1990s. In 1993 Patz et al. [3] showed the capability of [^18^F]FDG PET to differentiate benign from malignant focal pulmonary abnormalities detected on chest radiographs in a cohort of 51 patients: all the 18 benign lesions had an uptake of 2.5 or less. Few years later, the same group prospectively comparing visual assessment and SUV in distinguishing benign from malignant solitary pulmonary nodules, found that visual analysis was more sensitive but less specific than SUV [4]. Later, thousands of publications contributed to affirm SUVmax as the easiest and the most significant parameter to inform about diseases in both oncological and non-oncological settings. As a result, SUVmax has been introduced as a surrogate marker of aggressiveness, and a SUVmax of 2.5 has entered in practice as the cut-off to non-invasively characterise lesions, regardless of their size and site. Although, the nuclear medicine community has largely warned not to misuse this “easy” number, sensitive to factors related to scanners, image acquisition and reconstruction protocols, and patients [2, 5–8], it remains, especially among clinicians, one of the most requested and used parameters in PET imaging.

Lung lesions are common, and despite prevention and awareness campaigns, global regulations on tobacco sale and use, and screening and follow-up programs for smokers the projected trend of lung cancer incidence—one of the most costly and deadly tumors [9] —will continue to rise over the next 20 years [10]. Both CT and [^18^F]FDG PET/CT are recommended in the workup of a solitary lung nodule [11, 12]. Size and growth rate are key factors in assessing the malignant potential of a lung nodule, as the likelihood of malignancy positively correlates with its diameter. The role of morphology should not be underestimated [13]. Nomograms and AI-based tools have been also developed to assist imagers in determining the risk of malignancy [14–16]. However, despite correlations between certain CT features, [^18^F]FDG uptake patterns, and histology [17, 18], imaging-based diagnosis of lung lesions remains challenging.

This paper intend to provide robust evidence about the role of the SUVmax in a large cohort of subjects with suspected malignant lung nodules imaged by [^18^F]FDG PET/CT. Accordingly, our primary aim was to assess the diagnostic utility of SUVmax in distinguishing between benign, primary lung cancer subtypes and metastases. Secondary objectives were the a) assessment of factors that influence SUV measurements; b) evaluate the impact of different reconstruction protocols on SUV computation. Finally, we aimed at assessing SUVmax prognostic value. Additionally, our objective was to discuss and highlight the critical need to understand the limitations associated with parameters, scales, and references in imaging techniques.

## Materials and methods

### Population

In this retrospective single-centre study, we selected from an internal registry those patients that fulfilled the following inclusion criteria: a) a suspected or pathologically confirmed primary lung tumour; b) surgically treated, c) who underwent staging [^18^F]FDG PET/CT within 180 days before lung surgery; d) age > 18 years old, e) retrievability of [^18^F]FDG PET/CT images from the institutional PACS, and f) availability of pathology for the removed nodule (i.e. location and size of nodule, histological classification, and – if applicable – pathological staging). The exclusion criteria were a) incomplete [^18^F]FDG PET/CT data, and b) suboptimal quality of PET/CT images due to partial extravasation of [^18^F]FDG during the intravenous injection or blood glucose level > 200 mg/dL. For all patients we collected demographics, surgical information, histology, and follow-up data (as detailed in Supplementary material). Pathology was used as reference standard for the primary aim. Clinical outcome (relapse vs no evidence of disease) was used for survival analyses. We did not consider a minimum follow-up period. Patients after surgery were managed according to good clinical practice. The institutional Ethics Committee approved the study (approval number 3/18, on 17 April 2018, protocol ADVIMAG-Thorax).

### [^18^F]FDG PET/CT image acquisition and analysis

PET/CT images were acquired after the intravenous injection of 350–550 MBq of [^18^F]FDG, in accordance with the European Association of Nuclear Medicine (EANM) guidelines version 2.0 [19], as detailed in the supplementary Table 1. SUVmax and SUVmean of each lung lesion was calculated using the commercial software PET VCAR (GE Healthcare, Waukesha, WI, USA). Lesion segmentation was performed applying the adaptive threshold included in the PETVCAR software. If images reconstructed according to the EARL programme were available, SUVmax and SUVmean were recorded twice, with the clinical reconstruction and with the EARL programme protocol, respectively. For each patient we also collected scan date, injected activity, uptake time, and scanner (P1: Siemens Biograph 6 LSO, Siemens, Erlangen, Germany; P2: General Electric Discovery 690, General Electric Healthcare, Waukesha, WI, USA; and P3: Siemens Vision, Siemens, Erlangen, Germany).

### Statistical analysis

According to our primary aim, sample size calculation was based on sensitivity [20]. Literature data reported a prevalence of adenocarcinoma of 55% [21]. Fixing the predetermined value of specificity at 85% and keeping the maximum marginal error of estimate lower than 5% with 95% confidence level, the total number of required patients was 546 It was increased to 567 patients considering the possibility of a different frequency of adenocarcinoma in our population, non-primary lung cancer histology as well as negative cases.

Frequency tables and ratios were used to summarise the population’s general characteristics. According to histology, lung nodules were classified as benign, primary lung tumours (i.e. adenocarcinoma, squamous cell carcinoma, and other histological subtypes) or metastases. In case of multiple nodules, we considered all those pathologically assessed. The Wilcoxon rank sum test and Kruskal–Wallis test with multiple test correction were used to test differences between non-parametric data.

To evaluate correlations between SUVmax and potential influencing factors (patient weight, injected [^18^F]FDG activity, uptake time, histology, scanner, lung lesion size, neoadjuvant treatment) a multivariate linear regression analysis was performed.

Bland–Altman was used to compare the impact of clinical and EARL reconstruction on parameters quantification.

Survival analyses included only patients affected by primary lung tumour. Disease-free survival (DFS) and overall survival (OS) rates were analysed by Kaplan–Meier curves. A log rank test (Mantel-Cox) was run to compare survival curves. Cox proportional-hazards regression was used to test the association between survival and demographic, clinical, histological, and imaging data. Variables that resulted significant at univariate analysis and established prognostic factors were selected for multivariate Cox regression analysis. Since stage calculated according to the 7th and the 8th edition of the TNM classification could constitute a bias, we decided to include the size of the primary tumour and the N status in multivariate analysis. P-values < 0.05 were considered statistically significant. Stata MP (StataCorp LP, TX, USA) software, version 14.0 was used to perform statistical analyses.

## Results

### Population

Applying the above-mentioned criteria, we selected 567 patients, studied from 2014 to 2023. Primary lung tumour was diagnosed in 516/567 cases; 23/567 nodules resulted metastases from other tumours and 28/567 lesions were benign (including hamartomas, intraparenchymal lymph node, sarcoidosis, amyloid nodule, haemangiomas, abscess, sclerocalcific nodule). Table 1 summarises the main characteristics of the population. Supplementary Table 2 details the stage according to histology of primary lung tumour. Follow-up data on disease outcome and survival were available for all 516 patients with primary lung tumour (Supplementary Table 3). At last follow-up (median follow-up time of 27.53 months), 371 patients resulted with no evidence of disease. While disease relapse (median time of 13.54 months) was observed in 145 patients. Fifty-six patients died during follow-up.

**Table 1:** Main characteristics of the population (N = 567)

Demographics		
Age	Mean 59 ± 9.64 years	
	Median 65 (range 25–85) years	
Sex (M:F)	322:245	
Weight	Mean 70.69 ± 13.63 kg	
	Median 70 (range 41–120) kg	
[^18^F]FDG PET/CT		
Time from scan to surgery	Mean 85.5 ± 32.24 days	
	Median 63 (range 0–180) days	
Scanner	P1 = 258	
	P2 = 282	
	P3 = 27	
Injected activity	Mean 353.58 ± 15.63 MBq	
	Median 352 (range 303–423) MBq	
Uptake time	Mean 62.03 ± 7.82 min	
	Median 61 (range 42–102) minutes	
	< 55 min = 76	
	55–75 min = 454	
	> 75 min = 37	
EARL reconstruction	Yes = 166 (available only for P1 and P2)	
	No = 401	
Surgery		
Neoadjuvant chemotherapy	Yes = 77	
	No = 490	
Side of lesion	Left lung = 232	
	Right lung = 335	
Type of surgery	Open = 384	
	VATS = 110	
	RATS = 74	
Extent of resection	Lobectomy = 483	
	Bilobectomy = 9	
	Pneumonectomy = 28	
	Segmentectomy = 37	
	Resection = 11	
Pathology		
Classification	Benign lung lesion = 28	
	Malignant primary lung lesion = 516	
	• Adenocarcinoma = 361	
	• Squamous cell carcinoma = 93	
	• Carcinoid = 26	
	• Other = 36	
	Metastatic lung lesion = 23	
	• Colon = 14,	
	• Kidney = 3	
	• Melanoma = 2	
	• Breast = 1	
	• Pancreas = 1	
	• Uterus = 1	
	• Thyroid = 1	
Size of lung lesion	Mean 3.18 ± 2.18 cm	
	Median 2.6 (range 0.3–15.9) cm	
pT	**TNM v7**	**TNM v8**
	ypTis = 1	ypTis = 1
	pT1a = 17	pT1a = 19
	ypT1a = 2	ypT1a = 4
	pT1b = 19	pT1b = 73
	ypT1b = 3	ypT1b = 9
	pT1c = 1	pT1c = 61
		ypT1c = 9
	pT2a = 32	pT2a = 80
	ypT2a = 9	ypT2a = 13
	pT2b = 12	pT2b = 25
		ypT2b = 4
	pT3 = 20	pT3 = 52
	ypT3 = 2	ypT3 = 8
	pT4 = 1	pT4 = 21
	ypT4 = 2	ypT4 = 9
	NA = 59	
pN	N0 = 347	
	N1 = 65	
	N2 = 98	
	Nx = 1	
	NA = 58	
Lung Tumour Outcome (n = 516)		
Recurrence		
Yes	145 (28%)	
No	371 (72%)	
Progression-free survival		
Mean ± SD Median, IQR	678 ± 690 days 412, 86–1151 days	
Status at last follow-up		
Alive	460 (89%)	
Dead	55 (11%)	
Overall survival		
Mean ± SD	826 ± 737 days	
Median, IQR	655, 113 – 1383 days	

*IQR *interquartile range, *NA* not available; *RATS* Robotic-Assisted Thoracoscopic Surgery; *SD* standard deviation, *VATS* video-assisted thoracoscopic surgery

### [^18^F]FDG PET/CT imaging results

**Fig. 1:**
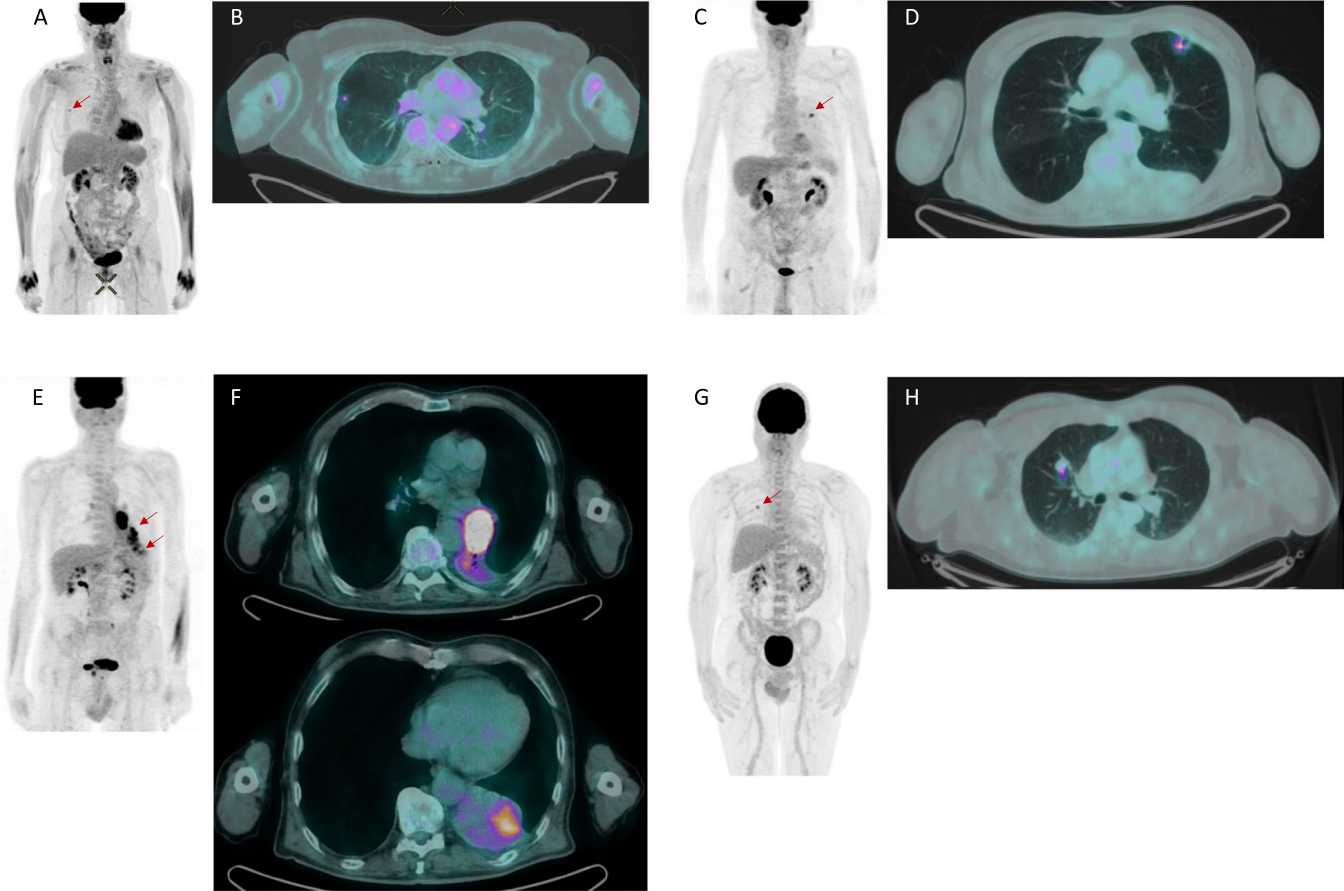
Clinical examples of different pattern of [.^18^F]FDG uptake in patients with suspected lung nodules. MIP and axial fused PET/CT of four different patients diagnosed with a benign intraparenchymal lymph node (**a**, **b**), primary lung adenocarcinoma (**c**, **d**), primary lung squamous cell carcinoma (**e**, **f**), and a lung metastasis from melanoma (**g**, **h**)

**Fig. 2:**
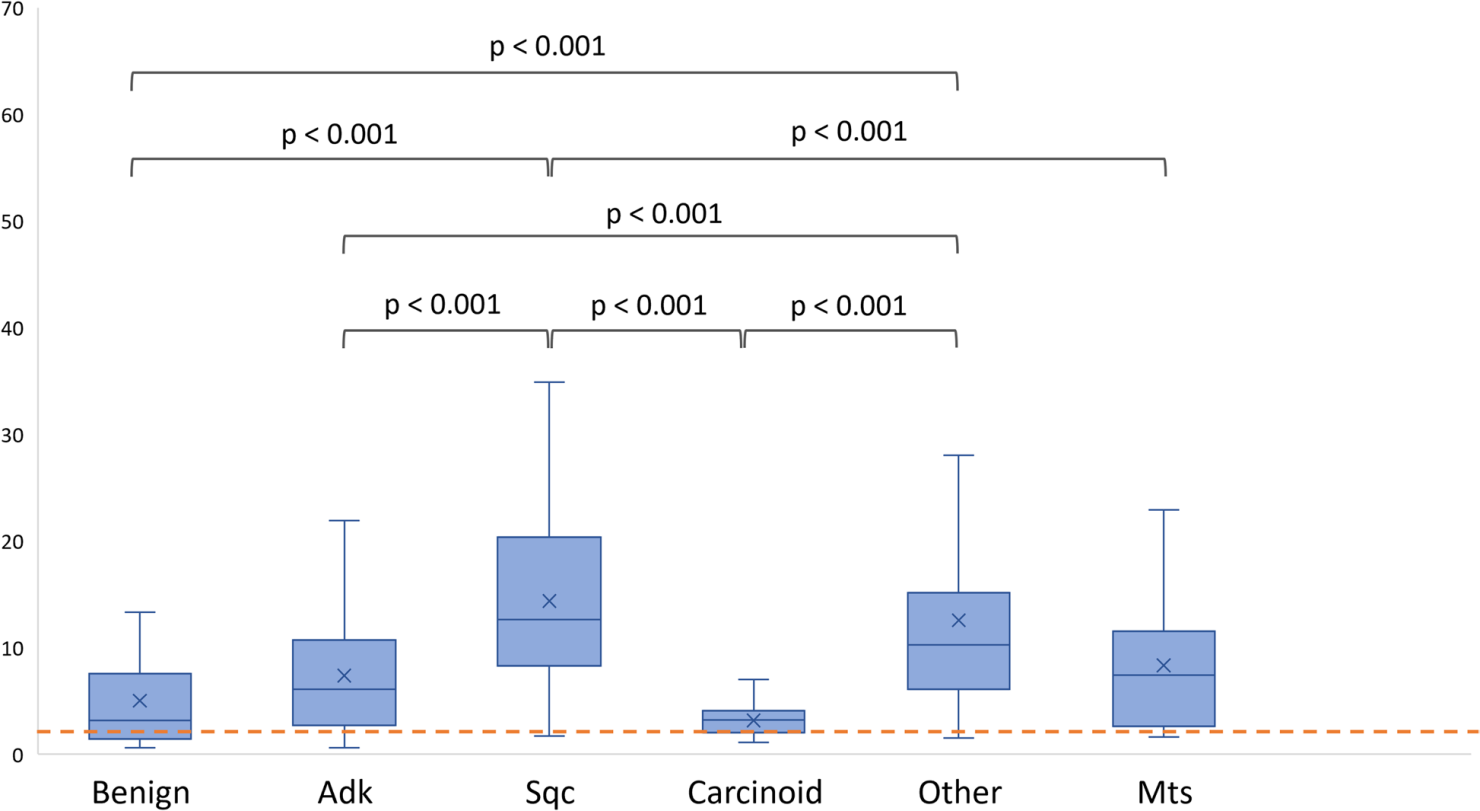
distribution of SUVmax according to histology (only significant correlation are reported). The light red line coincides with the threshold of 2.5

Values of SUVmax and SUVmean according to histology are detailed in Supplementary Table 4. Figure 1 shows some clinical examples. Benign nodules had SUVmax comparable to Adk (*p* = 0.886), carcinoids (*p* = 1.000), and metastases (*p* = 0.996). SUVmax did not differ between Adk and metastases (*p* = 1.000), between Sqc and other primary lung histology (*p* = 1.000), between carcinoids and metastases (*p* = 0.069), and between “other” primary lung histology and metastases (*p* = 0.194). Equivalent results were achieved for SUVmean. Figure 2 shows distribution of SUVmax according to histology.

SUVmax and SUVmean of patients with stage I disease were significantly lower than values of patients with stage II or III (*p*-value < 0.001 for both). SUVmax and SUVmean did not significantly differ among PET/CT scanners (p-value 0.14 for both). Table 2 summarises data on SUVmax and SUVmean according to TNM stage and scanner. At multivariate linear regression analysis (Table 3), SUVmax significantly correlated with patients’ weight (p-value 0.027), injected [^18^F]FDG activity (*p*-value 0.041), and lung lesion size (*p*-value < 0.001). Neoadjuvant therapy presented a negative trend with SUVmax, without reaching statistical significance (*p*-value 0.063). The overall model is statistically significant and has a good fit to the data (F(11, 499) = 32.24, *p* < 0.001).

**Table 2:** SUVmax and SUVmean according to TNM stage, and scanner

	**SUVmax**	*p***-value**	**SUVmean**	*p***-value**
**TNM stage**		< 0.001*		< 0.001*
* Stage I*	5.2 (2.3 – 9.7)		2.6 (1.6 – 4.7)	
* Stage II*	9.3 (4.4 – 15.1)		4.4 (2.4 – 8)	
* Stage III*	9.1 (5.4 – 13.9)		4.5 (2.5 – 7)	
**Scanner**		0.14		0.14
* P1*	7.2 (3 – 12.5)		3.5 (1.8 – 6)	
* P2*	6.75 (3.2 – 12.1)		3.2 (1.8 – 6)	
* P3*	3.7 (2 – 9.5)		2 (1.3 – 5)

**Table 3:** Multivariate linear regression analysis

Variable	β coefficient	95% CI	*p*-value
Weight	0.043	0.005 – 0.081	0.027*
[^18^F]FDG injected activity	−0.034	−0.07 – −0.014	0.041*
Uptake time	0.016	−0.04 – 0.08	0.603
Scanner			
* P1*	1 (Reference)		
* P2*	0.42	−0.56 – 1.4	0.4
* P3*	−0.62	−3.2 – 1.96	0.64
Histology			
*Benign*	1 (Reference)		
*Adk*	−1.64	−12.5 – 9.2	0.76
*Sqc*	3.4	−7.5 – 14.3	0.54
*Carcinoid*	−5	−16 – 6.07	0.37
*Other*	3.99	−7.03 – 15	0.47
Size of lung lesion	1.61	1.37 – 1.85	< 0.001*
Neoadjuvant therapy	−1.29	−2.6 – 0.68	0.063
Constant	12.4	−3.4 – 28.2	0.124

### Impact of reconstruction on SUVmax and SUVmean

EARL reconstruction was available in 166/567 patients (benign nodules *n* = 5, adenocarcinoma *n* = 116, squamous cell carcinoma *n* = 28, carcinoid *n* = 9, “other” histology *n* = 7, and lung metastasis from breast cancer *n* = 1). Sixty-four out of 166 patients were acquired with P1 and 102/166 with P2. SUVmax and SUVmean differed significantly between clinical and EARL reconstruction (*p*-value < 0.001 for both).

According to primary tumour histotype, there was a statistically significant difference in patients with adenocarcinoma (*p*-value 0.012 and 0.008, respectively) and squamous cell carcinoma (*p*-value 0.005 and 0.004, respectively). Reconstruction significantly affected SUVs in stage I patients (*p*-value < 0.001 for both SUVmax and SUVmean). Clinical and EARL reconstruction SUVs differed between scanners (*p*-value < 0.001 for both P1 and P2; Supplementary Fig. 1).

Table 4 summarizes data on SUVmax and SUVmean calculated according to clinical reconstruction and EARL, respectively. The Bland Altman plots for the difference between clinical and EARL reconstruction for SUVmax and SUVmean are displayed in Supplementary Fig. 2. The mean difference in SUVmax values between clinical and EARL reconstruction was 0.67 (95%CI −3.19 – 4.54), and for SUVmean was 0.32 (95%CI −1.65 – 2.3).

**Table 4:** SUVmax and SUVmean results according to reconstruction protocol

	**SUVmax**			**SUVmean**		
	**Clinical**	**EARL**	*p***-value**	**Clinical**	**EARL**	*p***-value**
**Total**	6.85 (3 – 12.6)	6.4 (2.4 – 12.3)	< 0.001*	3.3 (1.8 – 6.4)	3.1 (1.5 – 5.8)	< 0.001*
**Histology**						
* Benign*	1.4 (0.9 – 9.7)	1.5 (0.9 – 11.4)	0.18	0.9 (0.6 – 3.7)	0.9 (0.7 – 4.1)	0.5
* Adk*	6.35 (2.7 – 11.5)	5.8 (2.25 – 10.3)	0.012*	3 (1.7 – 6)	2.8 (1.3 – 5.2)	0.008*
* Sqc*	15 (8.2 – 22.5)	13.3 (6 – 21)	0.005*	6.8 (3.5 – 10.5)	5.8 (3.2 – 11)	0.004*
* Carcinoids*	3.1 (2.9 – 3.4)	2.8 (1.9 – 3.9)	0.84	1.9 (1.7 – 2.2)	1.6 (1.3 – 2.1)	0.19
* Other*	11.5 (5.9 – 28)	11.2 (4.4 −23.7)	0.81	5.2 (2.9 – 15)	5.2 (2 – 12.1)	0.68
* Mts*	8.2	6	1.0	4.2	2.9	1.0
**Stage**						
* Stage I*	5 (2.1 – 8.7)	4.2 (1.7 – 7.6)	< 0.001*	2.45 (1.3 – 3.7)	2 (1.2 – 3.8)	< 0.001*
* Stage II*	10.6 (4.9 – 16.3)	9.8 (4 – 17)	0.29	5.2 (2.2 – 8.7)	4.4 (2.1 – 8.9)	0.19
* Stage III*	11.3 (6.3 – 15.5)	9.9 (6 – 13.4)	0.044*	5.5 (2.8 – 7.3)	5.1 (2.8 – 7.1)	0.07
**Scanner**						
* P1*	7.3 (4.6 – 13)	8.5 (5.5 – 14)	< 0.001*	3.6 (2.2 – 6.5)	4.2 (2.3 – 7.2)	< 0.001*
* P2*	6.7 (2.7 – 12.6)	4.6 (2 – 10.4)	< 0.001*	3.1 (1.7 – 6.3)	2.3 (1.2 – 5.2)	< 0.001*

### Survival analyses

The median DFS for the entire cohort was 86.7 months (IQR 16.6 – not reached). Patients who relapsed had a median DFS of 12.6 months (IQR 6–21). DFS was significantly higher in patients with early-stage disease compared to advanced disease (86 and 63 months for stage I and II vs. 19 months for stage III, p-value < 0.001; supplemental Fig. 3a) and in patients treated with minimally invasive approaches compared to open surgery (not reached for Video-Assisted Thoracoscopic Surgery – VATS – and Robot-Assisted Thoracoscopic Surgery – RATS – vs 51 months for open surgery, p-value 0.002; supplemental Fig. 3b). DFS differed according to neoadjuvant therapy (21 months for patients who underwent neoadjuvant therapy vs not reached, p-value < 0.001; supplemental Fig. 3c), but statistical significance was not reached by stratifying DFS according to disease stage (*p*-value 0.17; supplemental Fig. 3d).

The other variables were not able to stratify patients’ DFS. The results of univariate and multivariate regression analysis are described in Supplemental Table 5. The correlation between SUVmax and DFS found at univariate analysis was lost at multivariate analysis. The stage (i.e. primary tumour size and N status) was the only significant predictor of DFS at multivariate analysis.

The median OS for the entire cohort was 88 months (IQR 66.5 – not reached). Patients who relapsed had a median OS of 69 months (IQR 40 – 88) and patients who died had a median OS of 26 months (IQR 11 – 45). Supplementary Fig. 4 shows that OS was longer in patients with early-stage disease compared to advanced disease (88 months and not reached for stage I and II, respectively, vs 80 months for advanced stage, p-value 0.02), in patients with carcinoid compared to other lung cancer histotypes (88 months for carcinoids vs 77 vs 76 vs 57 for Adk, Sqc and “other” histotypes respectively, p-value 0.01), and in patients who did not experience disease recurrence (69 months for relapsed vs not reached for not relapsed, p-value 0.001). The other variables were not able to stratify patients’ OS. The results of univariate and multivariate analysis are detailed in Supplementary Table 6.

Histology and N status were significantly associated with OS at univariate analysis. At multivariate analysis, squamous cell histology, “other” histological subtypes, stage (i.e. primary tumour size and N status) and SUVmax were significantly associated with OS. The same results were obtained in the Cox regression analysis for both DFS and OS when only patients with adenocarcinoma and squamous cell carcinoma were included, excluding the less common histological classes (i.e., carcinoid and other histological subtypes).

## Discussion

Our findings confirmed, in a large cohort, the limits of the use of SUVmax. Indeed, SUVmax alone, resulted not statistically different between benign lesions and adenocarcinoma, lung carcinoids, and metastases (Fig. 2). This was expected since, on one hand, a number of benign diseases (e.g., haemangiomas, infections and inflammation) appearing as solid or in part solid nodules, can exhibit high [^18^F]FDG uptake [4, 22–24]. On the other hand, adenocarcinomas and especially ground-glass opacity, which could represent anadenocarcinoma in situ [25], typically present significantly lower SUVmax than squamous cell carcinomas and other aggressive lung cancer subtypes, yet significantly higher than carcinoids [26, 27]. Moreover, in small size lesions the SUVmax performs poorly in differentiating between benign and malignant lesions [28] also due to “partial volume effect”, as also recently confirmed by the SPUTNIK trial in which different optimal SUVmax cut-offs—from 1.75 for lesions < 12 mm to 3.6 for nodules > 16 mm—have been proposed according to the diameter [29].

SUVmax has been claimed as a strong diagnostic, predictive, and prognostic marker in many conditions [30–35]. Frequently, SUV values are mentioned in the scan reports and its use is becoming more and more common especially among clinicians [36]. Overall, it seems that the findings on SUV from pioneer papers published from Patz et al. [3, 4] were misinterpreted by users and readers. Indeed, the authors concluded in a prospective cohort study, that visual analysis may provide a more easily generalizable interpretation method, especially when dealing with small nodules (≤ 15 mm) [4]. SUV-based parameters lack of normal range. Different lesions and diseases can present heterogeneous uptake. Several factors can affect SUV calculation (e.g. lesion’s size and location, scanner, reconstruction protocol) [2, 37–44]. These data were confirmed by our results. We found that patient-related factors, lesion size and imaging protocols can influence SUV measurements (Tables 3 and 4). Moreover, a large variability has been reported also when considering the same tracer within a healthy tissue (e.g. heathy skeleton SUV values differs between bones [45]).

As for prognostic value of SUV-based measures, at multivariate analysis we found a correlation between SUVmax and OS (HR 0.88, p-value 0.001), but not between SUVmax and DFS. We confirmed that the most advanced the stage, the shorter the DFS. Additionally, we found that more aggressive tumour subtypes (i.e. squamous cell carcinoma and other lung cancer subtypes), which are characterized by higher SUVmax, were correlated with a poorer outcome (Supplemental Fig. 3 and 4) especially in advanced stages. Notably, neither the type nor the extension of surgery affected DFS and OS. Still out of the main focus of the present analysis, our findings confirmed the well-established prognostic factors in lung cancer related to epidemiology, different histotypes and staging [46, 47]. Consequently, we cannot confirm the independent prognostic value of SUVmax in lung cancer.

Even if SUVmax is a simple measurement of tracer uptake within a region of interest, the axiom “the most avid the lesion, the more its aggressiveness” is not true. Meyer et al. [48] evaluating the correlation between the expression of glucose-transporter 1 and 3 expression and SUV in different tumours, did not found a linear association between these parameters. Indeed, SUVmax lacks normal ranges, [^18^F]FDG is not specific, and exerted external stimuli may influence lesion’s uptake (e.g. the flair effect related to treatment).

The meta-analyses on the prognostic role of SUVmax in NSCLC published by the European Lung Cancer Working Party for the International Association for the Study of Lung Cancer Staging Project defined SUVmax as “potentially a very interesting factor for predicting patient outcome” [34, 35]. Nonetheless, results of individual studies included in the meta-analyses showed a high heterogeneity related to the huge variability in terms of populations, SUV threshold (from 2.5 to 15), and type of threshold (best cut-off, median, arbitrary, validation, unknown). Even when limiting the analysis to the most robust studies to reduce heterogeneity, they were unable to establish the independent prognostic value of SUV, nor to conclude to an optimal threshold [34, 35]. Certainly, study design and sample size might negatively affect results and their validation. Moreover, rather than a dichotomous threshold that separates patients into two groups, a continuous hazard risk should be considered, also taking into account the significant weight of histology. Lastly, the inherent limitations in SUV reproducibility cannot be ignored. SUVs differ between scanners and vendors making comparison among machines (analogic second-, third-, modern-generation, and digital) even more irreproducible [37, 43, 44]. Despite our analysis showed that semi-quantitative values were unaffected by scanner, probably because the sample size was sufficient to mitigate the noisy effect, a significant disparity was observed between clinical and EARL reconstructions (p-value < 0.001). Moreover, our results further strengthened the notion that reconstruction protocols affected SUV values [37, 39–42]. This highlights the profound impact these technicalities can have, regardless of the disease. Therefore, although successful harmonization programs have been proposed and put in place [49–53], in daily practice they are insufficient to generate fully reproducible SUV values.

Moreover, the significance of SUVs is dependent on the radiopharmaceutical and its biological properties, therefore values and thresholds used for [^18^F]FDG are not adequate for radiopharmaceuticals other than [^18^F]FDG.

Hodgkin lymphoma and vasculitis are among the success stories in the use of [^18^F]FDG uptake as a diagnostic and prognostic marker. In both settings, a scale based on visual assessment is used [54, 55]. Similarly, for theragnostic purposes the Krenning score and later its modified version is employed for patient selection in the setting of somatostatin receptor targeted radioligand treatment in neuroendocrine tumours [56]. Accordingly, the SUVmax absolute value derived from PET/CT scans is meaningless and visual interpretation only should be used.

The present study acknowledged some limitations. Firstly, it is retrospective and monocentric. However, we analysed the effect of different scanners and reconstruction protocols. Moreover, we did not test other SUV-derived parameters (such as SUV normalized for lean body mass—SUL), that are known to be less prone to variation than SUVmax [2, 57]. However, it was out of the scope of the present work since we aimed to assess the diagnostic utility of SUVmax, which is the most commonly utilized measure of [^18^F]FDG avidity in clinical practice. Secondly, our cohort, coherently with epidemiology, was mostly composed by adenocarcinomas. Nonetheless, the sample size was calculated considering this disproportion and increased to minimise the effect of other confounders. Thirdly, not all patients were staged using the same edition of the TNM classification. However, we replaced the stage with the size of each lesion and the N status. Finally, an in-depth analysis of epidemiological, surgical, and oncological insights derivable from our population were not possible due to the lack of relevant information including lifestyle, environmental, and genetic data. However, this was beyond the scope of the present study.

## Conclusion

In this large monocentric cohort study of surgically treated lung lesion patients, SUV was not able to discriminate between different histotypes of lung cancer, and notably was not capable of differentiating between benign and malignant lesions. Patients presenting with an advanced stage of disease demonstrated higher SUVmax and SUVmean values, but none of these parameters managed to predict independently survival. The inherent bond between SUV values and known factors directly determining it but bearing little-to-none clinical significance is to be expected. Nonetheless, we are not suggesting an abrupt abandonment of SUV and SUV-derived parameters in research or clinical practice, as SUV is still able to encapsulate in a single numerical value a rough estimate of in vivo relevant biological information (metabolic activity or receptor expression level). This biomarker proved its’ contribution in a number of clinical scenarios. Our goal is to raise awareness on the fact that, similarly to other biomarkers, SUV can be influenced by confounders. Consequently, PET reports should rely on visual interpretation and avoid a direct mention of rough SUV values. Not all that glitters is gold: there is not a magic number, it is just another piece of the puzzle.

## Key points

**Question**: Are SUV values reliable diagnostic and prognostic biomarkers in patients with suspicious lung nodules?

**Pertinent findings**: SUV values failed to effectively differentiate between benign lesions, different subtypes of primary lung cancer, and metastases. SUV values are significantly influenced by patient weight, injected [^18^F]FDG activity, lesion size, and reconstruction protocols. Survival analyses revealed no independent prognostic significance for SUVmax in progression-free survival. SUVmax correlated with overall survival, alongside disease stage and tumour histotype.

**Implications for patient care:** Influencing factors significantly affect SUV interpretation, highlighting the need for cautious consideration in clinical practice.

## Supplementary Information

Supplementary Material 1.

## Data Availability

The manuscript represents valid work, and neither this manuscript nor one with similar content under the same authorship has been published or is being considered for publication elsewhere. Arturo Chiti had full access to all the data in the study and takes responsibility for the data integrity and the accuracy of the data analysis. Raw data are available on specific request: 10.5281/zenodo.10567629.

## Abbreviations

SPECT: Single photon emission computed tomography
PET: Positron emission tomography
SUVmax: Maximum standardised uptake value
SUVmean: Mean standardised uptake value
EANM: European Association of Nuclear Medicine
Adk: Adenocarcinoma
Sqc: Squamous cell carcinoma
NET: Carcinoids
DFS: Disease-free survival
OS: Overall survival
VATS: Video-Assisted Thoracoscopic Surgery
RATS: Robot-Assisted Thoracoscopic Surgery
HR: Hazard ratio
IQR: Interquartile range
NSCLC: Non-small cell lung cancer

## Data availability

Raw data are available on specific request: 10.5281/zenodo.10567629.
