# Supplementary material for: Challenging the significance of SUV-based parameters in a large-scale retrospective study on lung lesions
Source: Cancer Imaging. 2024 Nov 26;24:162. doi: 10.1186/s40644-024-00807-3 (PMC11600847; doi:10.1186/s40644-024-00807-3)
Supplement: Supplementary file 1 — Supplementary Material 1. [file 40644_2024_807_MOESM1_ESM.docx]

**Supplementary material**

*Materials and methods*

Demographics included data about age, sex, and weight; Surgical information included date, type, and extension, histology included location and size of nodule, histological classification, and when applicable grading, molecular profile, and pathological staging according to the 7^th^ or the 8^th^ edition of the TNM classification. Follow-up data included date of recurrence if any, date/status at last follow-up.

*Tables and figures*

| **Supplementary Table 1: [^18^F]FDG PET/CT parameters for each scanner** | | | | | | |
| --- | --- | --- | --- | --- | --- | --- |
| **Acquisition parameters** | **Biograph –**  **Siemens (P1)** | | **Discovery 690 – General Electric (P2)** | | **Vision - Siemens (P3)** | |
|  | PET | CT | PET | CT | PET | CT |
| **^18^F-FDG activity (MBq)*** | 350–550 | – | 350–550 | – | 350–550 | – |
| **Min/bed position** | 2.5 | – | 2 | – | Flow-motion |  |
| **Crystal** | LSO | – | LYSO | – | LSO |  |
| **Reconstruction** | Iterative | – | Iterative, TOF Sharp IR | – | Iterative, Ultra HD | Iterative |
| **Matrix (pixels)** | 128×128 | 512×512 | 256×256 | 512×512 | 440 | 512 |
| **Resolution (mm)** | 5.3×5.3 | 0.98×0.98 | 2.73×2.73 | 1.37×1.37 | 1.65x1.65 | 0.6x0.6 |
| **Slice thickness (mm)** | 2.0 | 4.0 | 3.27 | 3.27 | 2.0 |  |
| **Slices** | – | 6 | – | 64 |  | 64 |
| **Voltage (kV)** | – | 130 | – | 140 |  | 120 |
| **Tube current (mA)** | – | 95 | – | 140 |  |  |
| **Clinical reconstruction** |  | – | Iterative, TOF Sharp IR | – | Iterative, TOF Ultra HD |  |
| *Administered activity was calculated according to the European Association of Nuclear Medicine (EANM) guidelines 2.0 [21] | | | | | | |

| **Supplementary Table 2: stage of primary lung tumors according to histology** | | | | |
| --- | --- | --- | --- | --- |
| **Histology** | Stage | | | |
|  | I (n, %) | II (n, %) | III (n, %) | Total (n, %) |
| **Adk** | 191, 53% | 65, 18% | 105, 29% | 361 |
| **Sqc** | 40, 43% | 32, 34% | 21, 22% | 93 |
| **Carcinoids** | 17, 65% | 8, 31% | 1, 4% | 26 |
| **Other** | 15, 42% | 11, 30% | 5, 14% | 36* |
| **Total** | 263, 51% | 116, 22% | 132, 26% | 516* |
| *For 5 patients the stage was not assessed | | | | |

| **Supplementary Table 3. Data on outcome according to the by primary tumour histology and stage** | | | | |
| --- | --- | --- | --- | --- |
| **Outcome** | **Adenocarcinoma (n=361)** | **Squamous cell carcinoma**  **(n=93)** | **Carcinoid (n=26)** | **Other**  **(n=36)** |
| **Recurrence, n (%)** | 106 (30%) | 29 (31%) | 1 (4%) | 9 (25%) |
| **Progression-free survival, d**  **Mean ± SD**  **Median, IQR** | 697 ± 692  446, 110-1184 | 586 ± 650  378, 72 - 927 | 728 ± 783  381, 23-1238 | 698 ± 720  391, 56 - 1299 |
| **Death, n (%)** | 34 (9%) | 14 (15%) | 1 (4%) | 7 (20%) |
| **Overall survival, d**  **Mean ± SD**  **Median, IQR** | 859 ± 739  719, 166 - 1431 | 705 ± 666  576, 86-1075 | 788 ± 869  381, 23-1386 | 815 ± 788  671, 65-1589 |
|  | **Stage I (n=263)** | **Stage II (n=116)** | **Stage III (n=132)** | **Na (n=5)** |
| **Recurrence, n (%)** | 48 (18%) | 42 (36%) | 52 (39%) | 3 (60%) |
| **Progression-free survival, d**  **Mean ± SD**  **Median, IQR** | 754 ± 715  545, 118-1271 | 771 ± 722  454, 151 - 1317 | 449 ± 554  230, 36 - 592 | 511 ± 529  356, 72 - 825 |
| **Death, n (%)** | 20 (8%) | 14 (12%) | 22 (16%) | 0 (0%) |
| **Overall survival, d**  **Mean ± SD**  **Median, IQR** | 838 ± 745  661, 126-1423 | 945 ± 742  842, 295-1498 | 687 ± 689  523, 41-1084 | 1133 ± 1004  1582, 72-1896) |

| **Supplementary Table 4: SUVmax and SUVmean according to histology** | | |
| --- | --- | --- |
|  | **SUVmax** | **SUVmean** |
| **Histology** |  |  |
| *Benign* | 3.15 (1.45 – 7.5) | 1.65 (1.2 – 3.3) |
| *Adk* | 6.1 (2.7 – 10.6) | 2.9 (1.6 – 5.3) |
| *Sqc* | 12.6 (8.4 – 20.2) | 5.9 (3.7 – 10.2) |
| *Carcinoids* | 3.2 (2.1 – 4) | 1.9 (1.6 – 2.3) |
| *Other* | 10.25 (6.1 – 15) | 4.95 (3.05 -8.6) |
| *Mts* | 7.4 (2.6 – 11.5) | 3.4 (1.9 – 5.6) |

| **Supplementary Table 5: univariate and multivariate cox regression analysis for DFS** | | | | | |
| --- | --- | --- | --- | --- | --- |
|  | **Univariate analysis** |  | **Multivariate analysis** | | |
| **Variable** | **HR (95% CI)** | **p-value** | **HR (95% CI)** | | **p-value** |
| Age | 0.99 (0.97-1.00) | 0.336 | 0.99 (0.98 – 1.02) | 0.88 | |
| Histology |  | 0.016* |  |  | |
| *Adenocarcinoma* | 1 (Reference) |  | 1 (Reference) |  | |
| *Squamous cell carcinoma* | 1.24 (0.82 – 1.86) | 0.313 | 1.11 (0.67 – 1.8) | 0.69 | |
| *Carcinoid* | 0.13 (0.02 – 0.96) | 0.045* | 0.19 (0.03 – 1.41) | 0.105 | |
| *Other* | 0.87 (0.4 – 1.7) | 0.7 | 0.58 (0.25 – 1.38) | 0.223 | |
| Primary tumour size | 1.18 (1.1 – 1.26) | <0.001* | 1.13 (1.04 – 1.24) | 0.005* | |
| N descriptor |  | <0.001* |  |  | |
| *N0* | 1 (Reference) |  | 1 (Reference) |  | |
| *N1* | 2.1 (1.33 – 3.3) | 0.001* | 1.9 (1.18 – 3.05) | 0.008* | |
| *N2* | 2.86 (1.96 – 4.2) | <0.001* | 2.08 (1.35 – 3.2) | 0.001* | |
| *N3* | 15.14 (2.06 – 111.3) | 0.008* | 9.07 (1.07 – 76.83) | 0.043* | |
| SUVmax | 1.03 (1.01 – 1.05) | 0.006* | 1.00 ( 0.97 – 1.03) | 0.92 | |
| Neoadjuvant therapy *(no vs yes)* | 2.00 (1.35 – 2.94) | <0.001* | 1.46 (0.9 – 2.35) | 0.123 | |
| Surgical approach |  | 0.001* |  |  | |
| *Open surgery* | 1 (Reference) |  | 1 (Reference) |  | |
| *VATS* | 0.48 (0.29 – 0.78) | 0.003* | 0.71 (0.42 – 1.22) | 0.215 | |
| *RATS* | 0.52 (0.28 – 0.97) | 0.041* | 0.79 (0.41 – 1.51) | 0.472 | |
| Type of surgery |  | 0.14 |  |  | |
| *Lobectomy* | 1 (Reference) |  | 1 (Reference) |  | |
| *Bilobectomy* | nr |  | nr |  | |
| *Pneumonectomy* | 2.57 (1.34 – 4.9) | 0.004* | 1.01 (0.46 – 2.21) | 0.982 | |
| *Segmentectomy* | 1.04 (0.54 – 2.00) | 0.91 | 1.18 ( 0.59 – 2.36) | 0.634 | |
| *Resection* | 0.6 (0.08 – 4.32) | 0.615 | 0.79 (0.11 – 5.83) | 0.821 | |

| **Supplementary table 6: univariate and multivariate cox regression analysis for OS** | | | | | |
| --- | --- | --- | --- | --- | --- |
|  | **Univariate analysis** |  | **Multivariate analysis** | | |
| **Variable** | **HR (95% CI)** | **p-value** | **HR (95% CI)** | **p-value** | |
| Age | 1.01 (0.98-1.04) | 0.43 | 1.04 (1.00 – 1.08) | | 0.014* |
| Histology |  | 0.027* |  | |  |
| *Adenocarcinoma* | 1 (Reference) |  | 1 (Reference) | |  |
| *Squamous cell carcinoma* | 2.07 (1.1 – 3.87) | 0.023* | 5.0 (2.3 – 10.7) | | <0.001* |
| *Carcinoid* | 0.32 (0.04 – 2.46) | 0.274 | 0.2 (0.01 – 3.15) | | 0.26 |
| *Other* | 2.18 (0.96 – 4.94) | 0.061* | 10.8 (3.9 – 29.4) | | <0.001* |
| Primary tumour size | 1.1 (0.97 – 1.25) | 0.145 | 1.15 (0.98 – 1.36) | | 0.089 |
| N descriptor |  | <0.001* |  | |  |
| *N0* | 1 (Reference) |  | 1 (Reference) | |  |
| *N1* | 2.4 (1.17 – 4.95) | 0.017* | 1.7 (0.78 – 3.7) | | 0.183 |
| *N2* | 3.4 (1.9 – 6.2) | <0.001* | 4.36 (2.14 – 8.9) | | <0.001* |
| *N3* | nr |  | nr | |  |
| SUVmax | 0.99 (0.95 – 1.03) | 0.66 | 0.9 (0.84 – 0.97) | | 0.004* |
| Neoadjuvant therapy *(no vs yes)* | 1.77 (0.96 – 3.24) | 0.066 | 0.82 (0.38 – 1.77) | | 0.62 |
| Surgical approach |  | 0.35 |  | |  |
| *Open surgery* | 1 (Reference) |  | 1 (Reference) | |  |
| *VATS* | 0.65 (0.32 – 1.35) | 0.25 | 1.48 (0.65 – 3.36) | | 0.34 |
| *RATS* | 0.62 (0.22 – 1.7) | 0.36 | 0.78 (0.26 – 2.36) | | 0.66 |
| Type of surgery |  | 0.43 |  | |  |
| *Lobectomy* | 1 (Reference) |  | 1 (Reference) | |  |
| *Bilobectomy* | nr |  | nr | |  |
| *Pneumonectomy* | 2.08 (0.74 – 5.8) | 0.16 | 2.8 (0.84 – 9.5) | | 0.093 |
| *Segmentectomy* | 1.57 (0.65 – 3.76) | 0.31 | 2.08 (0.8 – 5-53) | | 0.14 |
| *Resection* | nr |  | nr | |  |
| Disease relapse *(no vs yes)* | 4.6 (2.6 – 8.3) | <0.001* | 5.36 (2.8 – 10.36) | | <0.001* |


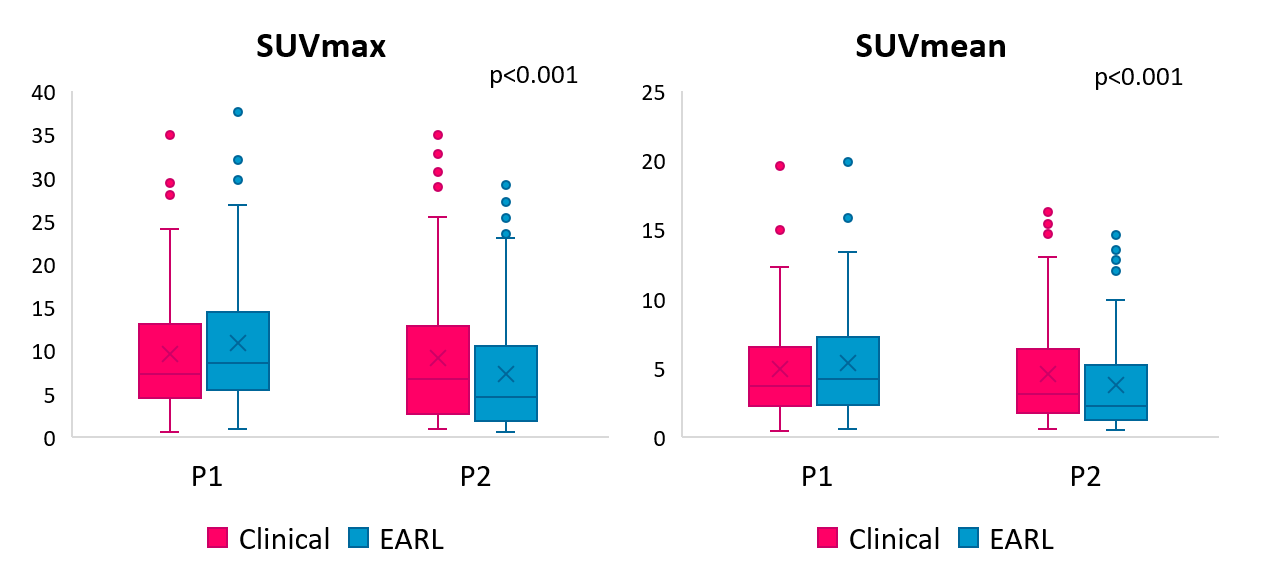


**B**

**A**

Supplementary Figure 1: box-plots of clinical and EARL SUVmax (a) and SUVmean (b) calculated for each scanner.


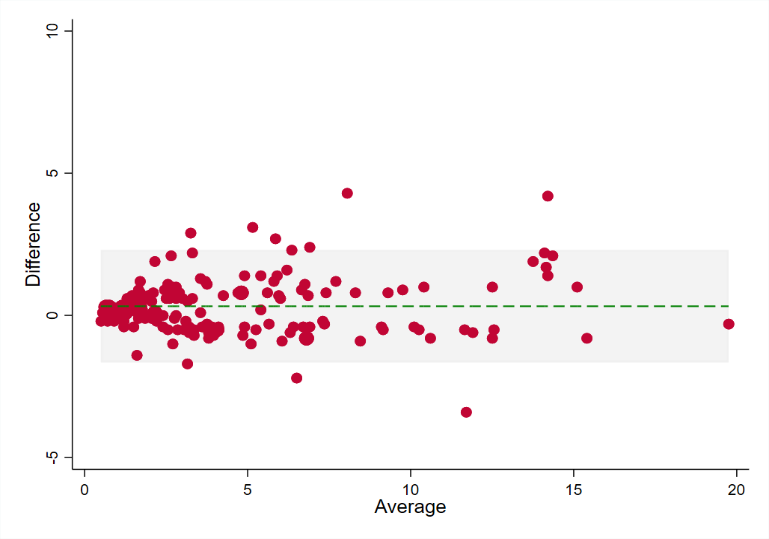

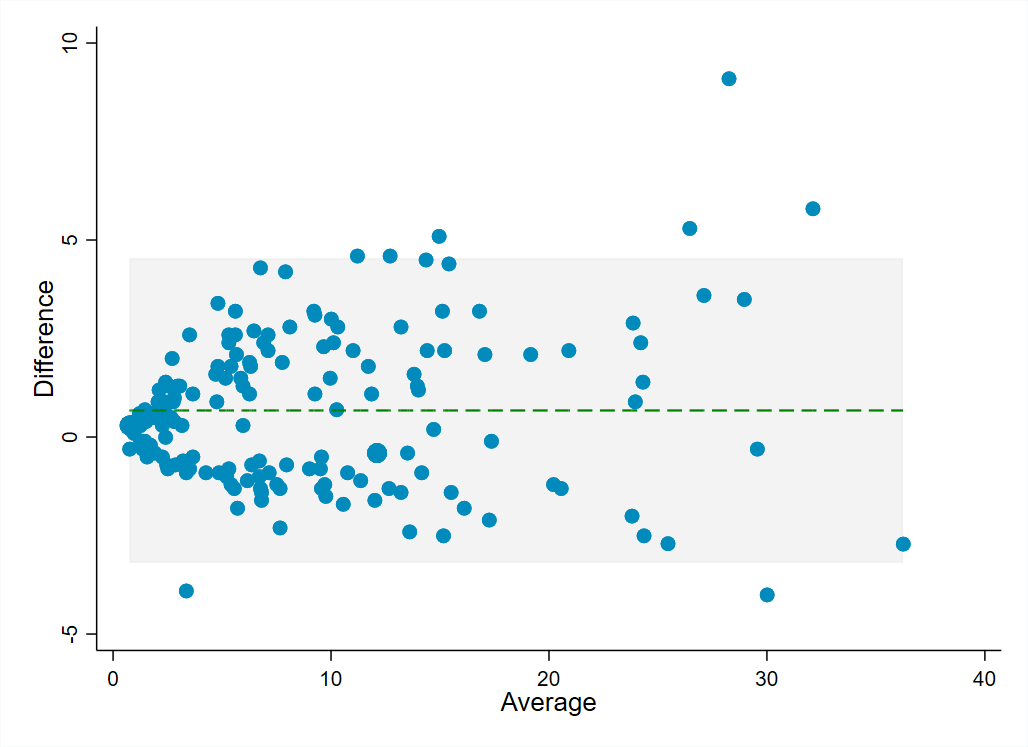


**A**

**B**

Supplementary Figure 2: Bland Altman plot for SUVmax (A) and SUVmean (B) .


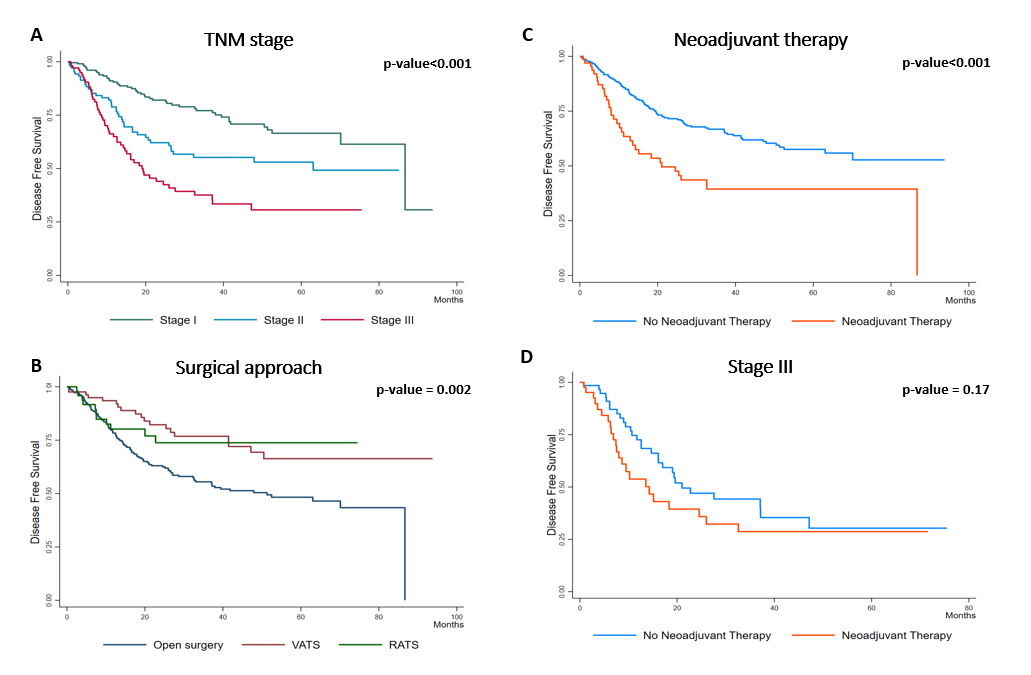


Supplementary Figure 3: Kaplan Meyer disease free survival estimate according to TNM stage (A), surgical approach (B), and neoadjuvant neoadjuvant therapy in the overall population (C) and in patients with advanced disease (D).


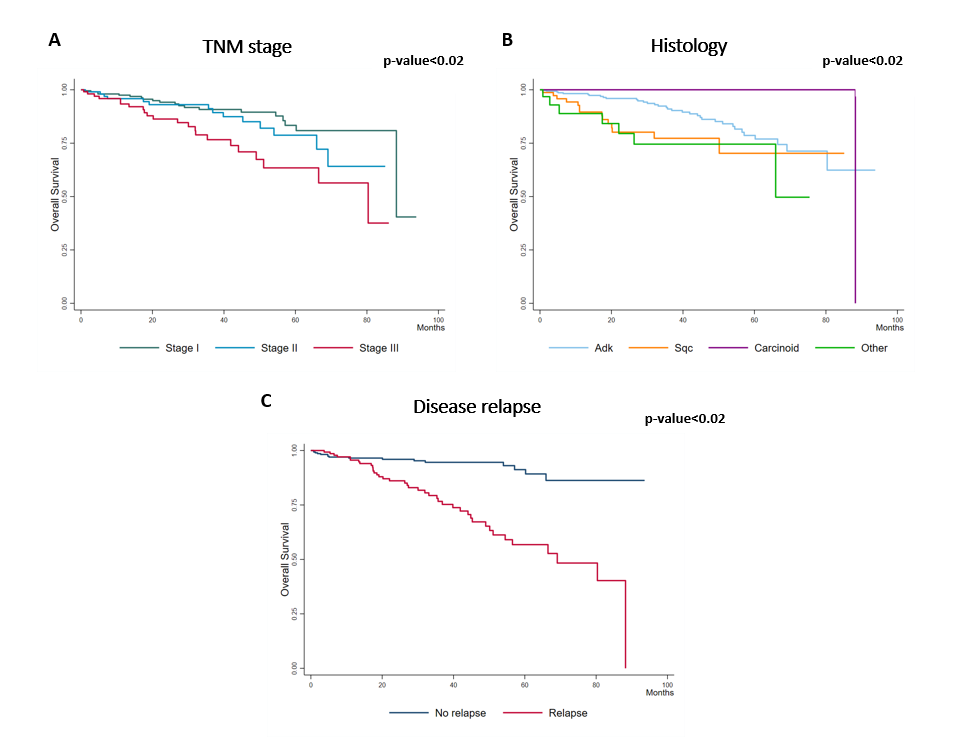


Supplementary Figure 4: Kaplan Meyer overall survival according to TNM stage (A), histology (B) and relapse (C).
